# Fault Mechanism Analysis of MEMS Gyroscopes Under High-Overload Environment

**DOI:** 10.3390/mi17101206

**Published:** 2026-10-21

**Authors:** Bin Yan, Zhongyang Guo, Yilong Chen, Jiantao Zhao, Yujun Yang, Shugang Zhang, Boyu Hao, Huiliang Cao

**Affiliations:** 1 Xi’an Microelectronics Technology Institute, Xi’an 710018, China; yanbin0991@126.com (B.Y.); cyil.019@163.com (Y.C.); zhaojiantao7@126.com (J.Z.); yanbin0991@hotmail.com (Y.Y.); xiebo771@126.com (S.Z.); 2 Anhui XDLK Microsystem Corporation Limited, Bengbu 233300, China; zyguo@numems.com; 3 School of Instrument and Electronics, North University of China, Taiyuan 030051, China; haoboyu123123@163.com; 4 School of Integrated Circuits and Electronics, Beijing Institute of Technology, Beijing 102488, China

**Keywords:** MEMS gyroscope, high-overload environment, parameter jump, mechanism analysis

## Abstract

High-overload micro-electro-mechanical system (MEMS) gyroscopes can retain an output after impact while exhibiting degraded sensing performance. This study examines a packaged four-mass gyroscope using a coupled mechanical model, transient finite element analysis and a single-device high-overload test. The transient model yields a peak stress of 181.412 MPa in the sensitive structure. The reported drive and sense frequencies change from 7734.0 and 7755.7 Hz to 7745.2 and 7751.3 Hz, respectively, reducing the frequency split from 21.7 to 6.1 Hz. Under a calibrated impact of 16,190 g, the zero-rate output changes from 0.00178 to 0.06733°/s and the reported bias instability from 3.52 to 143.8°/h. A smaller frequency split therefore does not establish improved post-impact performance. For unchanged effective modal mass, the reported frequencies imply equivalent stiffness changes of approximately +0.290% and −0.113%. These estimates do not establish silicon plasticity or uniquely identify a package mechanism. The results are interpreted as a mechanism-oriented case study, with residual stress redistribution and boundary condition changes considered possible explanations requiring further physical verification. Population reliability and repeatability are outside the evidence provided by this single-device, single-event dataset.

## 1. Introduction

MEMS gyroscopes offer compact size and compatibility with integrated inertial measurement systems [1,2,3]. Their resonant response, calibration, and mode-matching behavior depend on structural and control parameters [4,5]. For devices exposed to severe mechanical environments, structural survival and retention of accuracy are distinct reliability requirements [2,6,7]. A device that continues to produce output after impact may still exhibit changes in zero-rate output and bias instability.

A four-mass differential configuration offers a route to common-mode rejection through balanced motion and differential sensing [8,9,10]. Its performance nevertheless depends on stiffness, damping, electrode and readout symmetry. Additional masses and coupling beams increase the number of parameters that can become mismatched. Consequently, this architecture should not be assumed to outperform all single-mass or ring-based structures in shock resistance or bias stability without comparable measurements.

Recent work has explored machine learning for MEMS structural design. Chen et al. [11] represented disk resonator gyroscope topologies as binary matrices and used deep reinforcement learning, with a convolutional neural network surrogate for finite element evaluation, to search the design space. Their reported 7120 topologies belong to that independent study; the four-mass device investigated here was not selected through this search. Temperature compensation [12,13] and signal-processing approaches address gyroscope drift and noise [14,15]. EMD denotes empirical mode decomposition, which adaptively separates a signal into intrinsic mode functions and a residual [16]. Such processing does not itself demonstrate recovery of a mechanically altered sensor.

This study examines parameter changes in a packaged four-mass MEMS gyroscope after a calibrated high-overload event. The coupled model describes changes in modal response and provides a framework for distinguishing transient elastic deformation from persistent effective parameter changes. The experimental evidence consists of one device and one impact event; it is used to examine possible mechanisms rather than to qualify population reliability. No artificial intelligence optimization was performed on the tested device.

## 2. Structural Design and Modal Analysis of the MEMS Gyroscope

**Figure 1:**
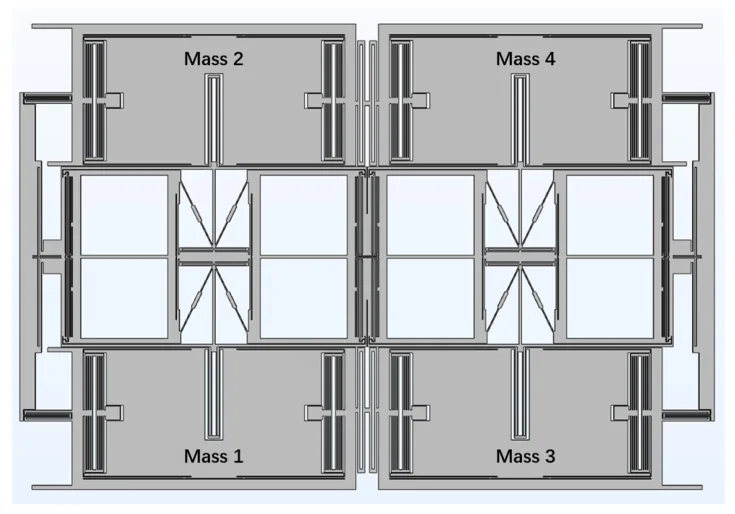
Finite element model of the four-mass MEMS gyroscope.

The sensitive structure of the MEMS gyroscope studied in this paper adopts a four-mass tuning fork symmetric structure. Due to the two-degree-of-freedom design, the entire four-mass structure involves many elastic beam connections, resulting in a relatively complex structure. The design of elastic beams makes their stiffness differ greatly in the drive and sense axis directions, which can be approximated as a spring-damper structure. The model of the structurally decoupled four-mass micro gyroscope is shown in Figure 1. The sensitive structure was supplied by Anhui XDLK Microsystem Corporation Limited. Some geometric and fabrication information is commercially confidential.

**Figure 2:**
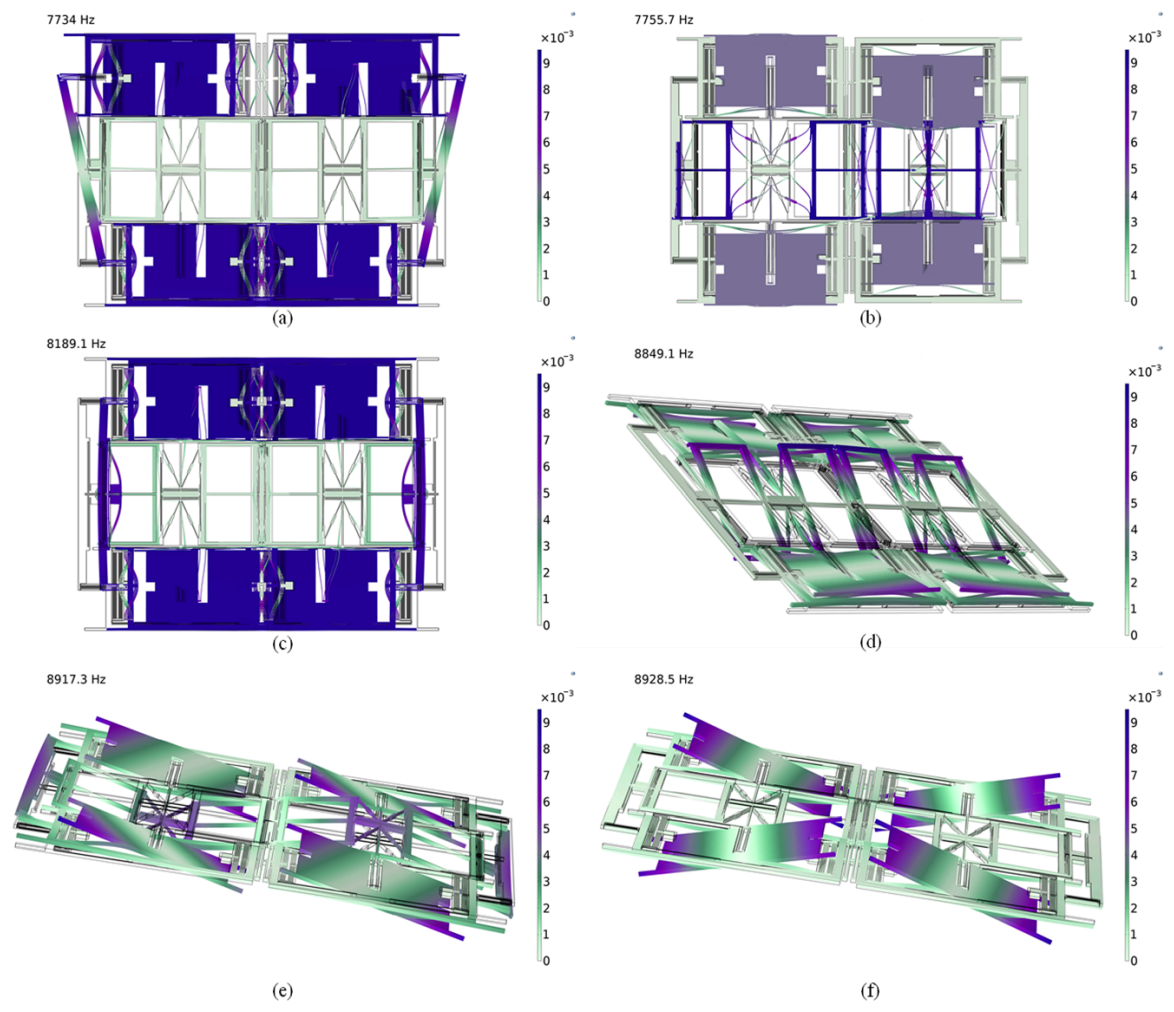
Working and parasitic mode shapes of the four-mass MEMS gyroscope. (**a**) First-order drive mode; (**b**) second-order sense mode; (**c**) third-order in-plane parasitic mode; (**d**) fourth-order out-of-plane parasitic mode; (**e**) fifth-order out-of-plane parasitic mode; (**f**) sixth-order out-of-plane parasitic mode.

The modal analysis of the four-mass MEMS gyroscope is shown in Figure 2. The frequency of the first-order mode (drive mode) is 7734.0 Hz, the frequency of the second-order mode (sense mode) is 7755.7 Hz, and the frequency of the third-order mode (in-plane interference mode) is 8189.1 Hz. The resonant frequencies of the fourth-order, fifth-order, and sixth-order modes (out-of-plane interference modes) are 8849.1 Hz, 8917.3 Hz, and 8928.5 Hz, respectively. The frequency separation between the working mode and other modes is greater than 400 Hz, which helps to reduce the influence from interference modes.

## 3. Fault Mechanism Analysis of the MEMS Gyroscope

**Figure 3:**
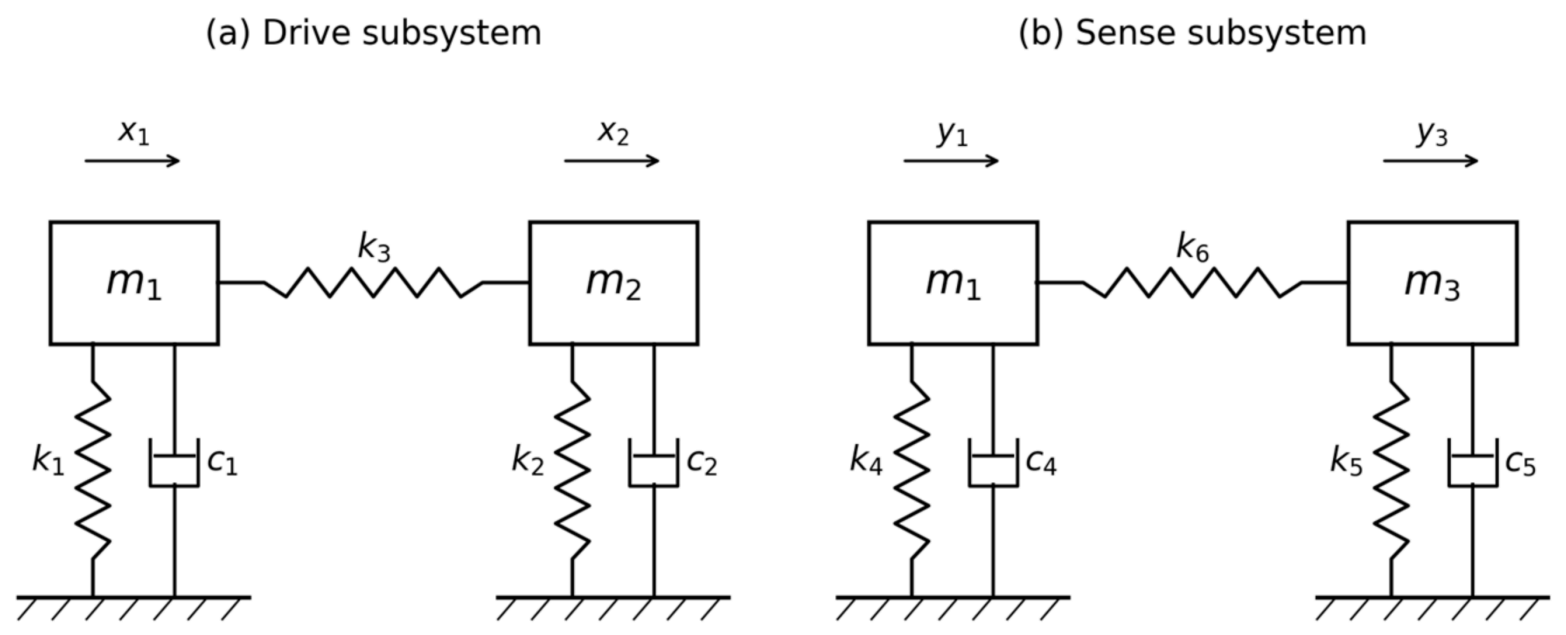
Reduced equivalent mechanical models: (**a**) drive subsystem; (**b**) sense subsystem. Coupling stiffnesses within each axis are distinguished from parasitic cross-axis coupling.

The four-mass device can be reduced to a pair of effective masses for drive mode analysis, with masses and frames lumped into *m*_1_ and *m*_2_. In Equations (1) and (2), *k*_1_ and *k*_2_ connect the masses to the supports, *k*_3_ connects the two masses, and *c*_1_ and *c*_2_ are damping coefficients. Figure 3 is a reduced mechanical representation rather than a fabrication drawing. The spring labels in the equivalent model must follow these definitions.

Figure 3 explicitly distinguishes the reduced drive and sense subsystems. In the sense subsystem, *m*_3_ is the effective mass associated with proof mass 3, *k*_4_ and *k*_5_ are support stiffnesses, *k*_6_ couples masses 1 and 3 in the sense direction, and *c*_4_ and *c*_5_ are their damping coefficients. The sense equations therefore introduce a different pair of coordinates rather than additional drive-mode degrees of freedom. In Equation (13), *β* is an effective stress-to-stiffness coefficient: its units are stiffness per stress, and its value depends on geometry, material, constraints, and stress direction. The relation is a local linearization; *β* has not been experimentally identified in this study.

In drive mode analysis, the following motion equations can be established by applying harmonic driving forces *F*_1_ = *F* sin *ωt* and *F*_2_ = −*F* sin *ωt* with opposite phases to proof masses 1 and 2:

(1) $$m_{1}\frac{d^{2}x_{1}}{dt^{2}}+(k_{1}+k_{3})x_{1}+c_{1}\frac{dx_{1}}{dt}=F\;sin\omega t+k_{3}x_{2}$$

(2) $$m_{2}\frac{d^{2}x_{2}}{dt^{2}}+(k_{2}+k_{3})x_{2}+c_{2}\frac{dx_{2}}{dt}=k_{3}x_{1}-F\;sin\omega t$$

This set of differential equations reveals the dynamic coupling mechanism between proof masses: the motion of each proof mass is not only restricted by its own inertial force, elastic restoring force, and damping force, but also produces mutual traction through the interconnection stiffness *k*_3_. To solve this binary second-order differential equation set, the complex vector method is introduced to simplify steady-state response analysis. Assuming that the system reaches a steady state under a driving force with frequency *ω*, the displacement of the proof mass and the driving force can be expressed as:

(3) $$x_{1}(t)=X_{1}e^{j\omega t},x_{2}(t)=X_{2}e^{j\omega t}$$

(4) $$F_{1}=-F_{0}e^{j\omega t},F_{2}=F_{0}e^{j\omega t}$$

Substituting the above expressions into Equations (1) and (2), the amplitude response of the proof mass can be derived through complex operations:

(5) $$X_{1}=\frac{-F_{0}[k_{2}+\omega (ic_{2}-m_{2}\omega )]}{[k_{2}+k_{3}+\omega (ic_{2}-m_{2}\omega )][k_{1}+k_{3}+\omega (ic_{1}-m_{1}\omega )]-k_{3}^{2}}$$

(6) $$X_{2}=\frac{F_{0}[k_{1}+\omega (ic_{1}-m_{1}\omega )]}{[k_{2}+k_{3}+\omega (ic_{2}-m_{2}\omega )][k_{1}+k_{3}+\omega (ic_{1}-m_{1}\omega )]-k_{3}^{2}}$$

When system parameters satisfy the symmetry conditions *m*_1_ = *m*_2_, *k*_1_ = *k*_2_, *c*_1_ = *c*_2_, amplitudes *X*_1_ and *X*_2_ present strict characteristics of equal amplitude and opposite phase.

For a constant input angular rate *Ω*, the Coriolis force is proportional to the drive velocity. With *x_j_*(*t*) = Re{*X_j_* exp(*iωt*)}, its force phasor *Q_j_* is given below, using a consistent sense-axis sign convention. This explicitly retains the 90° phase relation between displacement and velocity:

(7) $$Q_{1}=2i\cdot m_{1}\Omega \omega X_{1};\;\;Q_{3}=2i\cdot m_{3}\Omega \omega X_{3}$$

With *F_cj_*(*t*) = Re{*Q_j_* exp(*iωt*)}, the sense direction motion equations for proof masses 1 and 3 are:

(8) $$m_{1}\frac{d^{2}y_{1}}{dt^{2}}+(k_{4}+k_{6})y_{1}+c_{4}\frac{dy_{1}}{dt}=F_{c1}+k_{6}y_{3}$$

(9) $$m_{3}\frac{d^{2}y_{3}}{dt^{2}}+(k_{5}+k_{6})y_{3}+c_{5}\frac{dy_{3}}{dt}=F_{c3}+k_{6}y_{1}$$

Similarly, let the steady-state response of the sense mode be

(10) $$y_{1}(t)=Y_{1}e^{j\omega t},y_{3}(t)=Y_{3}e^{j\omega t}$$

Substituting into the equations, we can obtain:

(11) $$\begin{array}{c} Y_{1}=\frac{A_{3}Q_{1}+k_{6}Q_{3}}{A_{1}A_{3}-k_{6}^{2}}\\ A_{1}=k_{4}+k_{6}-m_{1}\omega^{2}+i\omega c_{4};\;A_{3}=k_{5}+k_{6}-m_{3}\omega^{2}+i\omega c_{5} \end{array}$$

(12) $$Y_{3}=\frac{k_{6}Q_{1}+A_{1}Q_{3}}{A_{1}A_{3}-k_{6}^{2}}$$

The ideal working state of the system depends on strict parameter symmetry. When the proof mass, elastic stiffness, and damping coefficient satisfy symmetry conditions, displacements of adjacent proof masses in the drive mode present perfect antisymmetric characteristics, and the sense mode output maintains a linear relationship with the angular velocity input. This symmetric design not only suppresses environmental interferences such as substrate acceleration, but also reduces the influence of temperature drift through common-mode rejection.

A nonuniform stress field can perturb support and coupling stiffnesses through geometric stiffness, strain, and boundary condition changes. For small perturbations, an effective local stress-to-stiffness relation can be written as:

(13) $$\Delta k_{i}=\beta \sigma_{i}$$

A stiffness perturbation can change eigenfrequencies and eigenvectors. Frequency proximity alone does not generate coupling in a perfectly decoupled system: energy transfer also requires a nonzero coupling term. Here *k*_3_ and *k*_6_ denote couplings within the drive and sense subsystems, respectively; they are not themselves drive-to-sense cross-stiffness terms. A separate parasitic cross-axis term is introduced in Equation (22). Stress may alter modal participation and effective modal mass, but the present data do not measure such a change.

This perturbation directly acts on the stiffness terms of Equations (1), (2), (8) and (9):

(14) $$m_{1}\frac{d^{2}x_{1}}{dt^{2}}+[(k_{1}+\Delta k_{1})+(k_{3}+\Delta k_{3})]x_{1}+c_{1}\frac{dx_{1}}{dt}=F\;sin\omega t+(k_{3}+\Delta k_{3})x_{2}$$

For the equal-mass drive subsystem, replacing *k*_1_, *k*_2_, and *k*_3_ with their perturbed values gives the undamped eigenfrequencies below. Define *a* = *k*_1_ + Δ*k*_1_ + *K*_3_ and *b* = *k*_2_ + Δ*k*_2_ + *K*_3_, where *K*_3_ = *k*_3_ + Δ*k*_3_:

(15) $$\omega_{\pm }^{2}=\frac{a+b\pm \sqrt{\left[(a-b{)}^{2}+4K_{3}^{2}\right]}}{2m}$$

Even when *k*_1_ = *k*_2_, the coupled two-mass subsystem generally has distinct in-phase and antisymmetric eigenfrequencies if *k*_3_ is nonzero. Equation (15) describes that within-axis splitting, not the measured drive–sense frequency split. Unequal perturbations also change the eigenvectors and may produce amplitude imbalance.

In Equations (11) and (12), stiffness terms *k*_4_, *k*_5_, and *k*_6_ are also affected by stress and become *k*_4_ + Δ*k*_4_, *k*_5_ + Δ*k*_5_, and *k*_6_ + Δ*k*_6_. At this time, the detection displacement response formula is rewritten as:

(16) $$\begin{array}{c} Y_{1}=\frac{B_{3}Q_{1}+K_{6}Q_{3}}{D_{s}}\\ D_{s}=[k_{4}+\Delta k_{4}+K_{6}-m_{1}\omega^{2}+i\omega c_{4}]\\ \times [k_{5}+\Delta k_{5}+K_{6}-m_{3}\omega^{2}+i\omega c_{5}]-K_{6}^{2}\\ K_{6}=k_{6}+\Delta k_{6};\;B_{1}=k_{4}+\Delta k_{4}+K_{6}-m_{1}\omega^{2}+i\omega c_{4}\\ B_{3}=k_{5}+\Delta k_{5}+K_{6}-m_{3}\omega^{2}+i\omega c_{5} \end{array}$$

(17) $$Y_{3}=\frac{K_{6}Q_{1}+B_{1}Q_{3}}{D_{s}}$$

The perturbed determinant in Equations (16) and (17) affects the amplitude and phase of the Coriolis response. Because *Q*_1_ and *Q*_3_ are proportional to angular rate, this model alone cannot produce a nonzero output at zero angular rate. A zero-rate error additionally requires parasitic drive-to-sense coupling, equilibrium or electrode asymmetry, or readout effects. These mechanisms are plausible but are not separately identified by the available measurements.

A stress-induced bending moment can produce a small tilt *θ*. An effective rotational oscillator represents its dynamic response, and the measured displacement may include a geometric contribution *lθ*:

(18) $$Y_{real}=Y_{1}+l\Theta ;\;\Theta =\frac{M_{\omega }}{k_{\theta }-J_{\theta }\omega^{2}+i\omega c_{\theta }}$$

Here *l* is the effective lever arm, *J_θ_* is the rotational inertia, *k_θ_* is the torsional stiffness, and *c_θ_* is the rotational damping coefficient. For a constant moment *M*_0_, the static tilt is *M*_0_/*k_θ_*; the frequency-domain expression describes an oscillatory moment. These quantities are illustrative effective parameters rather than independently measured properties of the present device.

A permanent length change of an ideal axial member changes its axial stiffness according to Equation (19). The expression fixes the length-change sign convention and is an illustrative constitutive branch, not a bending-beam model or evidence of silicon plasticity:

(19) $$\Delta k_{ax}=\frac{EA}{L_{0}+\Delta L}-\frac{EA}{L_{0}}\approx -\frac{EA\Delta L}{L_{0}^{2}}$$

Here *L*_0_ is the original length, Δ*L* is positive for elongation, *E* is Young’s modulus, and *A* is cross-sectional area. The approximation assumes |Δ*L*| ≪ *L*_0_ and fixed *E* and *A*. This idealized branch is not invoked to claim that silicon yielded in the present test. *A* purely elastic transient response cannot retain a post-load shift unless the state or constraints also change.

In Equations (20) and (21), Δ*k_i_*_,res_ denotes a persistent effective stiffness offset more generally. Candidate causes include changed residual preload or anchor/package constraints; these have not been separately verified. An asymmetric effective offset changes the drive response:

(20) $$\begin{array}{c} X_{1}\prime =\frac{-F_{0}[k_{2}+\Delta k_{2,res}-m_{2}\omega^{2}+i\omega c_{2}]}{D_{r}}\\ D_{r}=[k_{1}+\Delta k_{1,res}+K_{3}-m_{1}\omega^{2}+i\omega c_{1}]\\ \times [k_{2}+\Delta k_{2,res}+K_{3}-m_{2}\omega^{2}+i\omega c_{2}]-K_{3}^{2}\\ K_{3}=k_{3}+\Delta k_{3,res} \end{array}$$

(21) $$X_{2}\prime =\frac{F_{0}[k_{1}+\Delta k_{1,res}-m_{1}\omega^{2}+i\omega c_{1}]}{D_{r}}$$

At zero angular rate, parasitic cross-axis stiffness and damping can generate a drive frequency sense response. For a small parasitic coupling and prescribed drive motion, a reduced one-way response is:

(22) $$Y_{p}(\omega )=\frac{-(k_{xy}+i\omega c_{xy})X(\omega )}{k_{y}-m_{y}\omega^{2}+i\omega c_{y}}$$

In Equation (22), *k_xy_* and *c_xy_* are drive-to-sense cross-axis coefficients, while *m_y_*, *k_y_*, and *c_y_* are effective sense parameters. *Y*_p_ is an oscillatory displacement, not a static displacement. A phase-sensitive readout may convert it to an apparent rate offset. Thus, the zero-rate error requires parasitic coupling or other readout/equilibrium effects in addition to the Coriolis-only model; the present measurements do not identify their separate contributions.

## 4. Fault Simulation of the MEMS Gyroscope

The modeled assembly comprises gyroscope housing, the four-mass sensitive structure, bonded PCB, and potting adhesive. Transient finite element analysis was performed using COMSOL Multiphysics 6.3 to evaluate displacement and stress under an imposed impact load.

**Figure 4:**
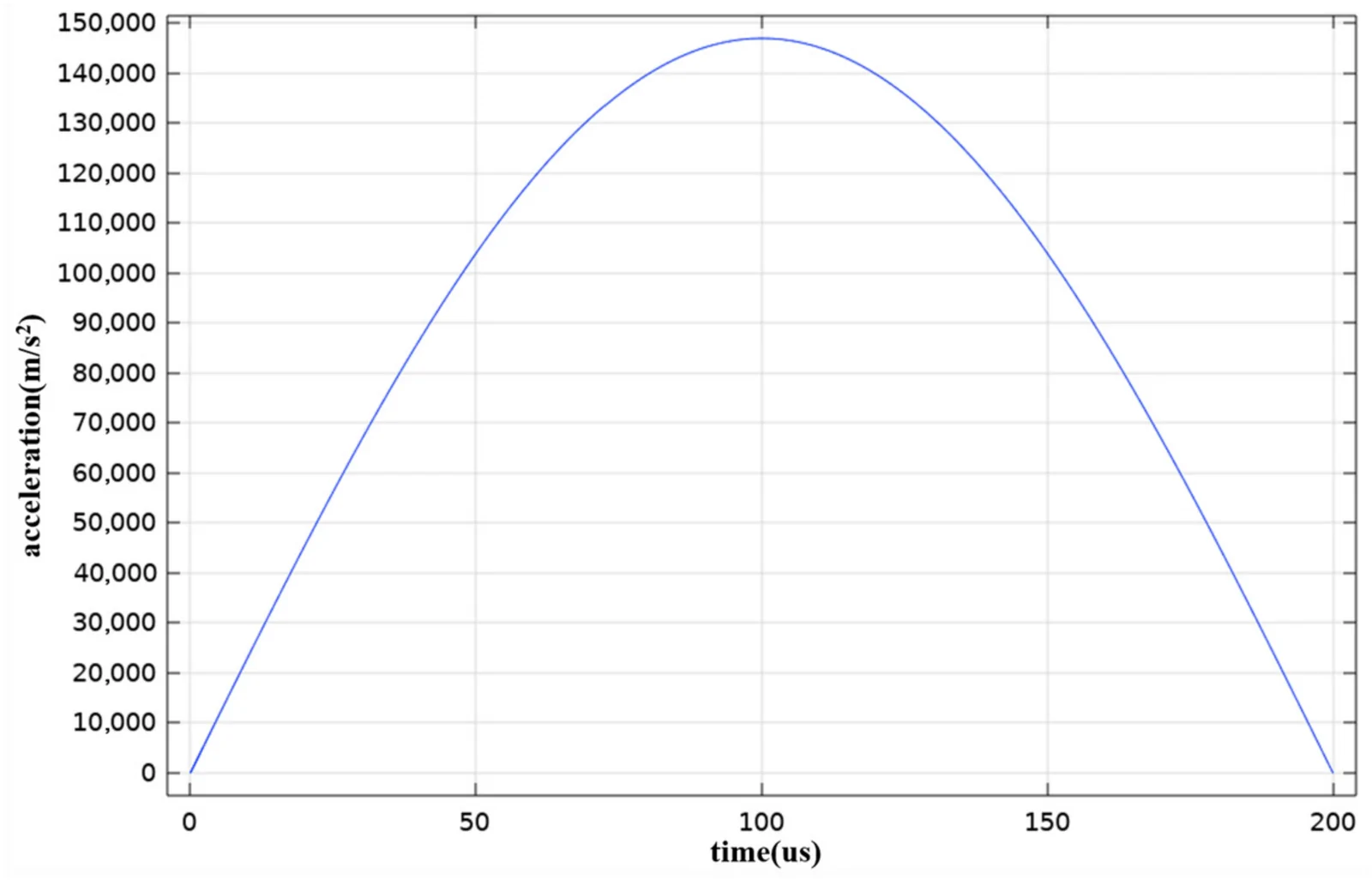
Impact function curve.

**Figure 5:**
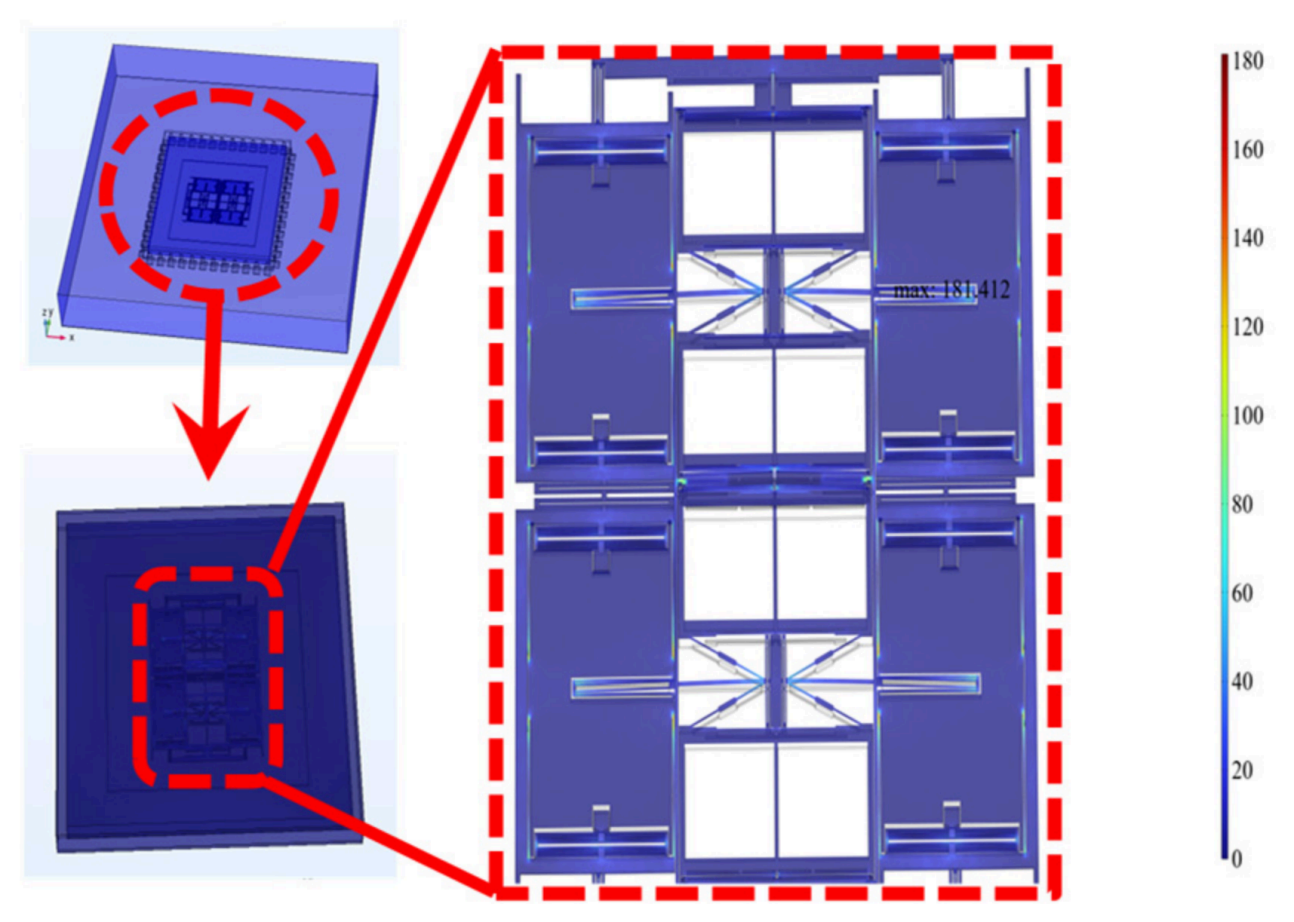
Assembly used in the impact simulation (unit: MPa).

**Figure 6:**
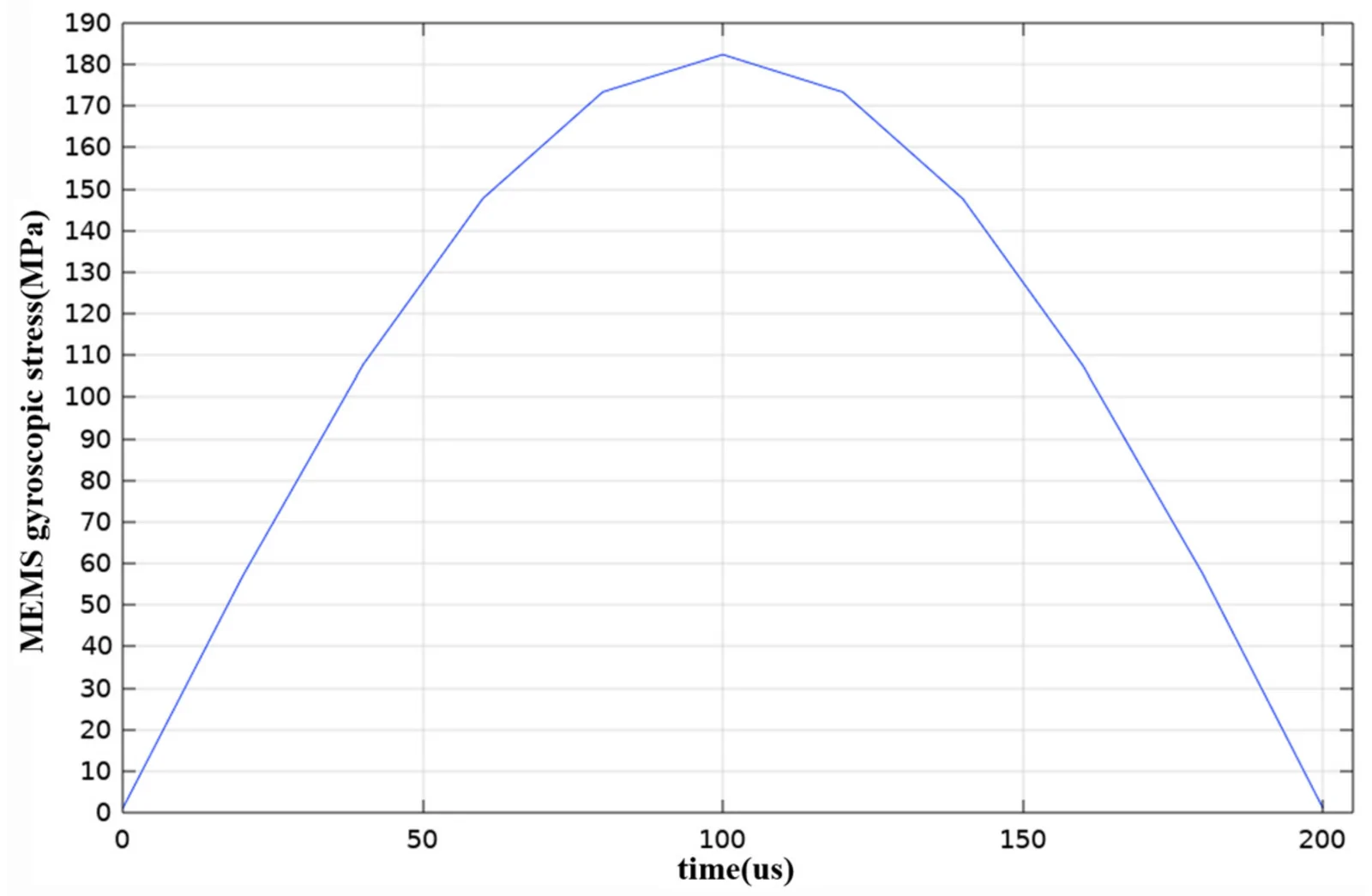
Maximum stress curve of impact simulation.

A half-sine pulse represents the imposed impact, as plotted in Figure 4. Figure 5 identifies the modeled assembly, and Figure 6 presents the computed peak stress history, whose reported maximum is 181.412 MPa.

The reported peak stress in the sensitive structure is 181.412 MPa. Relative to the nominal 790 MPa reference used in the original analysis, this is approximately 23.0%. That reference is not treated here as a universal silicon yield stress: silicon failure depends on stress criterion, defects, and geometry. This comparison does not prove that every package component or interface remains unchanged. A packaged device can, in principle, acquire a persistent effective parameter change through altered preload or constraints while its silicon resonator remains predominantly elastic. Packaging-induced frequency effects have been demonstrated in a thermally loaded resonant accelerometer [17]; this is analogous evidence for sensitivity to package stress, not proof of the shock mechanism in this gyroscope. The present model does not directly simulate or identify the post-impact residual state.

**Figure 7:**
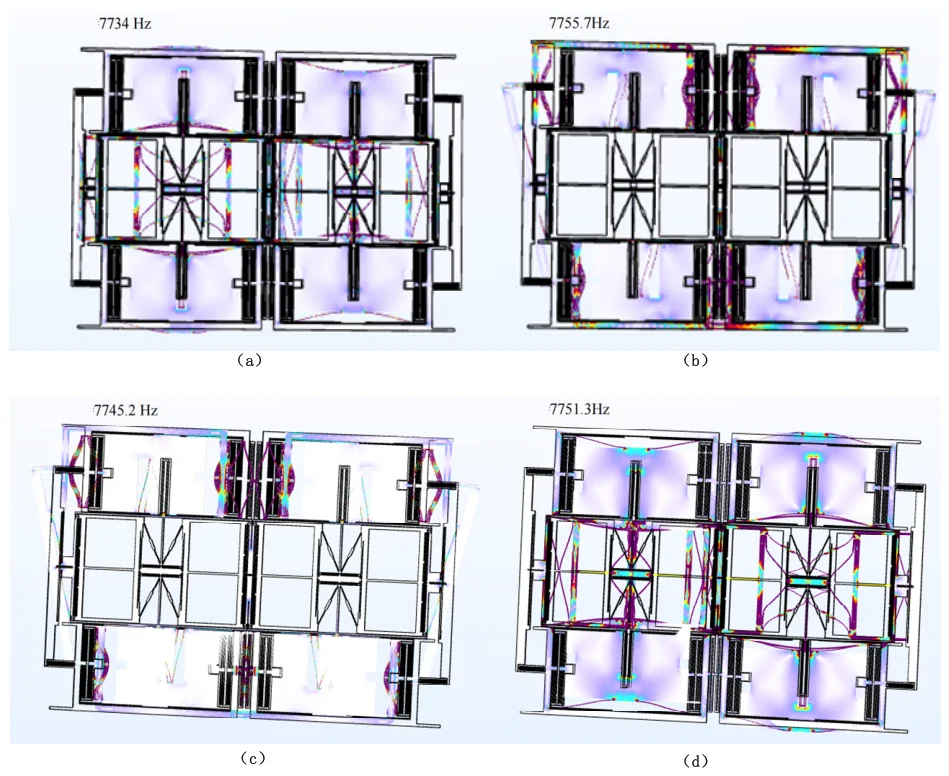
Changes in working mode frequencies of the four-mass MEMS gyroscope before and after impact. (**a**) Drive mode before impact; (**b**) sense mode before impact; (**c**) drive mode after impact; (**d**) sense mode after impact.

Figure 7 and Table 1 report drive frequencies of 7734.0 and 7745.2 Hz before and after impact, and sense frequencies of 7755.7 and 7751.3 Hz.

A smaller frequency split can be useful for mode matching [4], but the change from 21.7 to 6.1 Hz is not evidence of improved sensing performance. Frequency split is a scalar measure; it does not determine damping symmetry, modal-axis alignment, parasitic coupling, or readout response. In the reported dataset, the drive frequency changes by +11.2 Hz (+0.145%) and the sense frequency by −4.4 Hz (−0.057%), while zero-rate output and bias instability deteriorate. This establishes a coexistence of reduced split and degraded output metrics. It does not establish which unmeasured coupling or damping parameter changed.

## 5. High-Overload Experimental Verification

**Figure 8:**
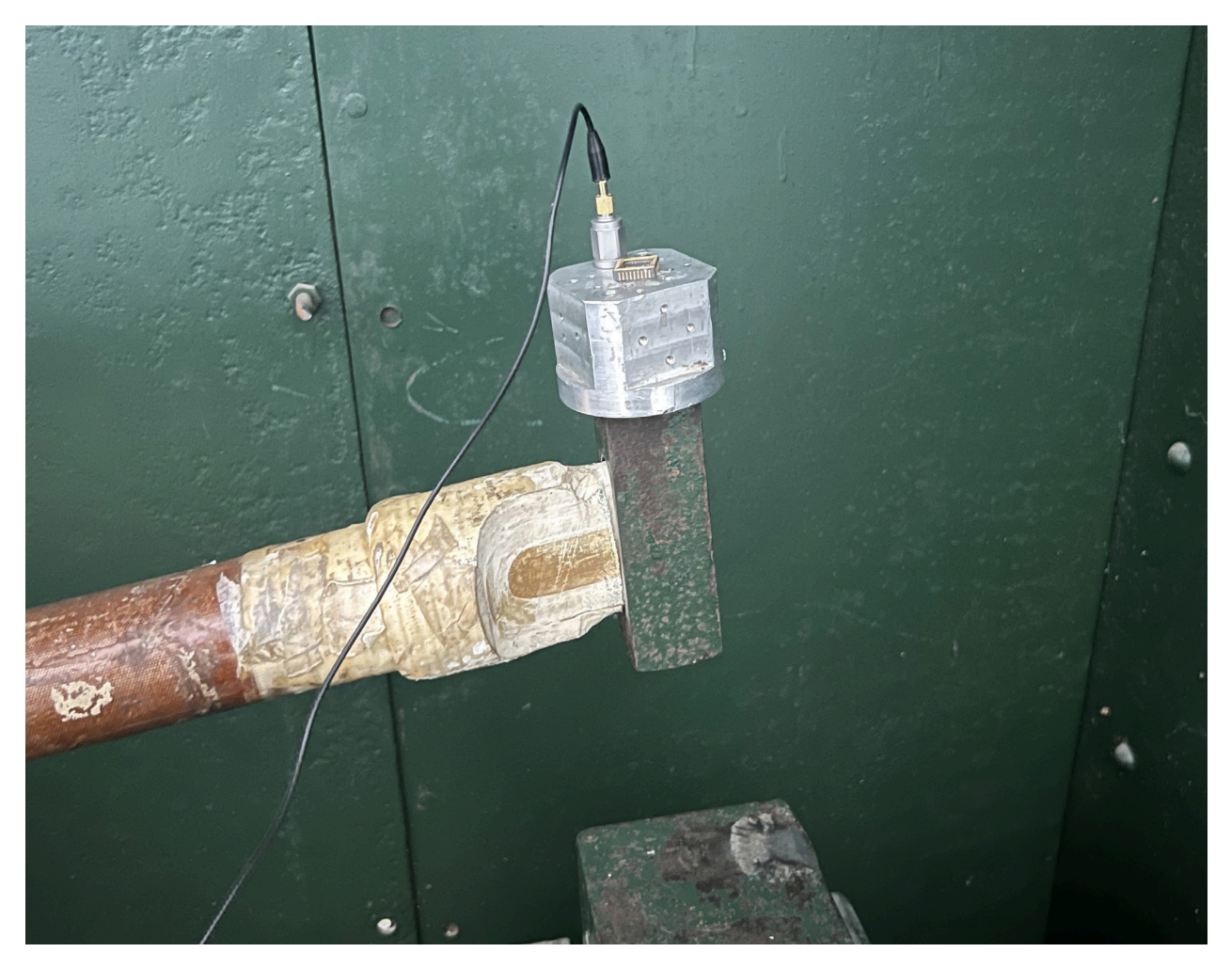
Installation diagram of the machete hammer.

**Figure 9:**
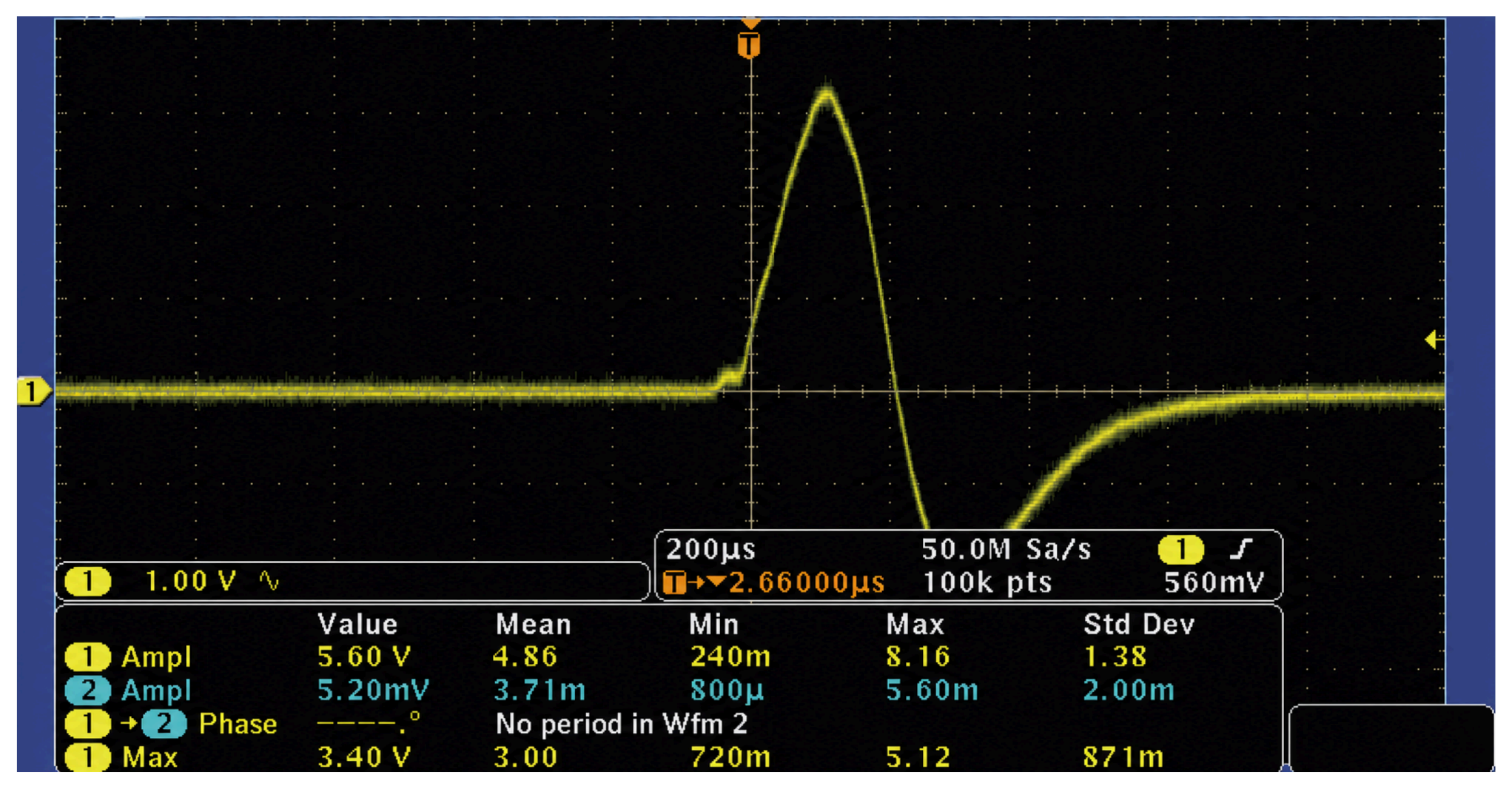
Calibrated impact response of the standard sensor used for overload pre-calibration.

**Figure 10:**
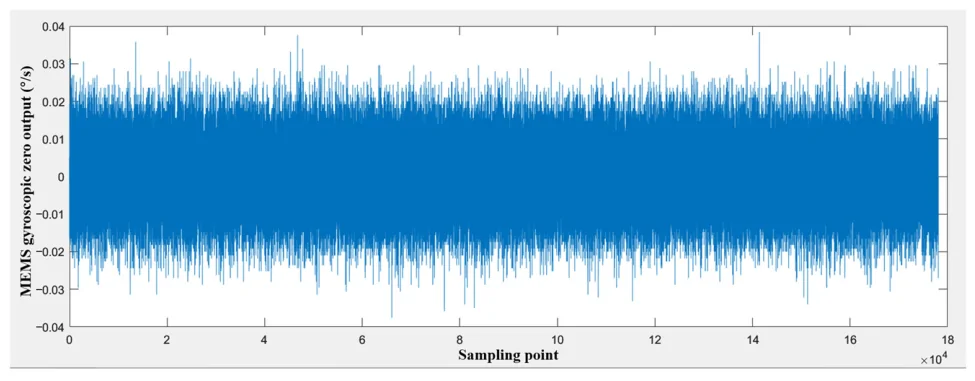
Zero-rate output of the MEMS gyroscope before impact.

**Figure 11:**
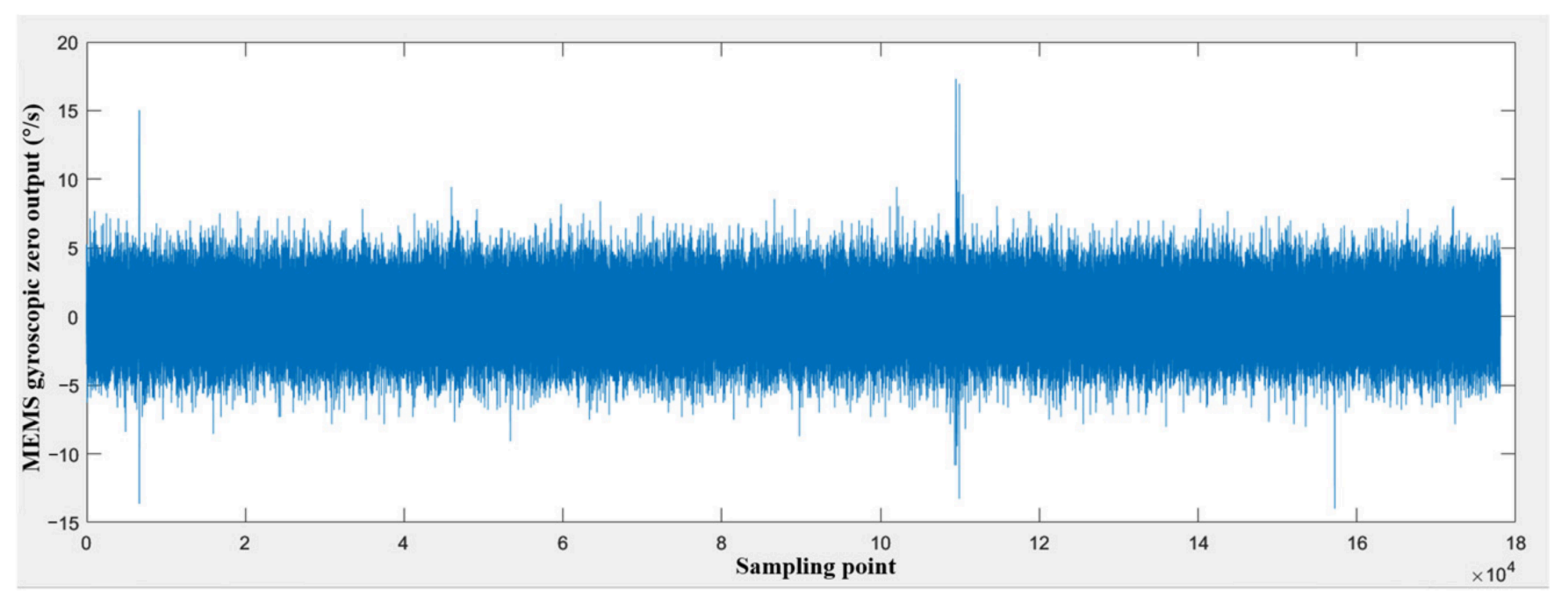
Zero-rate output of the MEMS gyroscope after impact.

The impact test is carried out on the four-mass MEMS gyroscope (Anhui XDLK Microsystem Corporation Limited, Anhui, China).using a machete hammer. First, a standard sensor is fixed on the hammer head for impact pre-calibration. The system sensitivity is measured as 0.21 mV/g through the signal chain composed of a charge amplifier and an oscilloscope. After obtaining the calibrated overload environment from the standard sensor response, the packaged gyroscope and the standard sensor are fixed on the hammer head for the formal impact test. After the test, the data are read and analyzed. Figure 8 shows the installation diagram of the machete hammer, and Figure 9 shows the representative impact response of the standard sensor used for pre-calibration. Figure 10 and Figure 11 show the zero-rate outputs of the four-mass MEMS gyroscope before and after impact, respectively.

**Figure 12:**
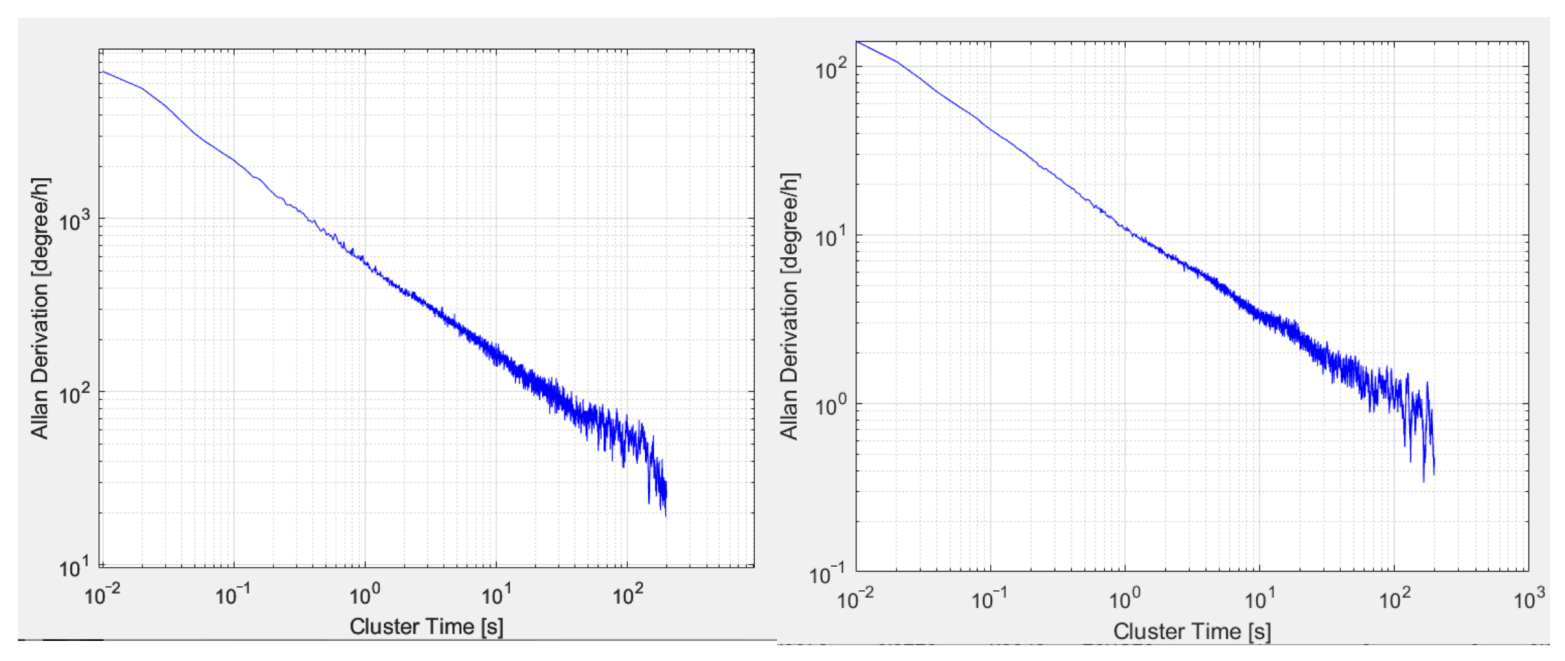
Allan variance curves of the four-mass MEMS gyroscope before and after impact.

The calibrated impact amplitude is reported as 16,190 g. The pre/post zero-rate outputs, converted using the turntable-calibrated scale factor, are 0.00178 and 0.06733°/s; their difference is 0.06555°/s from the reported rounded values. The reported Allan-deviation-based bias instability increases from 3.52 to 143.8°/h. Figure 10 and Figure 11 show the output records, and Figure 12 shows the Allan variance curves of the four-mass MEMS gyroscope before and after impact.

The device continued to produce an output after the reported impact, while its zero-rate output and reported bias instability changed markedly. Continued output is not proof that all mechanical components remained intact. This experiment is a mechanism-oriented case study of one packaged device subjected to one calibrated impact event. It cannot establish event-to-event repeatability, device-to-device variation, or a population failure probability. No additional physical experiments have been added. A controlled multi-device campaign, including the reviewer’s suggested 3–5 nominally identical devices as an initial repeatability assessment, and physical inspection of anchor/package interfaces remain necessary future work. The reported observables are consistent with parameter changes but do not uniquely establish a damage mechanism.

The results concern one packaged device and one impact event. Table 1 contains rounded summary values, not the information needed to estimate between-device or between-event repeatability or full measurement uncertainties. Assuming conventional rounding, half of the last displayed decimal increment provides a limited numerical reporting bound. This bound is not a standard or expanded measurement uncertainty; calibration, model, environmental, and Allan deviation estimation uncertainties remain unquantified.

## 6. Discussion and Conclusions

The reported frequency split decreases from 21.7 to 6.1 Hz, whereas the reported bias instability increases from 3.52 to 143.8°/h, a factor of approximately 40.9. Thus, smaller frequency separation is not a sufficient measure of post-impact performance. Modal-axis orientation, damping asymmetry, parasitic coupling, and the readout transfer function may also change. Published damping trimming work likewise treats damping asymmetry as a distinct error source [18]; this comparison does not establish that it caused the changes in the present device.

For unchanged effective modal mass, *f*^2^ ∝ *k*_eff_ gives the equivalent changes (7745.2/7734.0)^2^ − 1 ≈ +0.290% and (7751.3/7755.7)^2^ − 1 ≈ −0.113%. These estimates use the reported frequencies and describe effective modal parameters, not individual beam stiffnesses. They show that the observed frequency differences can be represented by small effective changes without requiring a hypothesis of silicon plasticity. They do not determine whether a package interface, residual stress, temperature, electrostatic tuning, or another factor changed. Establishing persistence also requires controlled measurement conditions and elapsed time information.

Comparison with other structures is therefore made at the mechanism level, alongside published shock reliability and protection studies [19,20]. The Quad Mass Gyroscope studied by Askari et al. [10] was characterized in open-loop and force-to-rebalance rate configurations, illustrating the importance of operation and control conditions. Lian et al. [21] examined tuning fork gyroscopes with displacement constraints and found that stops can alter shock response, including collision-induced failure. Neither study supplies a matched control for the present packaged device. Differences in pulse duration, direction, packaging, operating mode, and failure criterion preclude ranking shock resistance from peak acceleration alone. This study focuses on parameter degradation in a device that continues to produce output, rather than establishing a universal survival threshold.

The machine learning approach of Chen et al. [11] addresses disk resonator topology discovery; it was not used in the present experiments. Future application to impact-resistant four-mass designs could incorporate transient stress, collision clearance, and modal parameter sensitivity as additional objectives. Such a surrogate would need training data and validation for those objectives. No artificial intelligence optimization or optimal design claim is made for the present device.

This study combines a coupled mechanical model, transient simulation, and a single-device high-overload case study. The reported event changes the zero-rate output from 0.00178 to 0.06733°/s and the bias instability from 3.52 to 143.8°/h. Reduced drive–sense frequency separation therefore cannot be used alone to establish retained accuracy. The transient stress result and post-impact output metrics address different aspects of reliability. Residual preload or boundary condition changes are possible explanations for persistent effective parameter shifts, but the available evidence does not uniquely establish them or prove silicon plasticity. The conclusions apply to the reported device and event; controlled repeated testing and physical characterization are needed to establish reproducibility and causality. The precision-only intervals in Table 1 do not provide a complete measurement uncertainty assessment; in particular, they cannot resolve repeatability or establish the statistical significance of the pre/post changes.

## Figures and Tables

**Table 1:** Reported parameter changes before and after impact, with precision-only rounding bounds.

MEMS Gyroscope Parameters	Before Overload Test	After Overload Test
Drive mode frequency (Hz)	7734.0 ± 0.05	7745.2 ± 0.05
Sense mode frequency (Hz)	7755.7 ± 0.05	7751.3 ± 0.05
Zero position output	0.00178 ± 0.000005°/s	0.06733 ± 0.000005°/s
Zero bias instability	3.52 ± 0.005°/h	143.8 ± 0.005°/h

Note: The ± entries are half-last-digit rounding bounds only: ±0.05 Hz for frequencies, ±0.000005°/s for zero-rate output, and ±0.005/±0.05°/h for pre-/post-impact bias instability. They are NOT measured uncertainties, confidence limits, or repeatability estimates. Calibration, modeling, Allan deviation estimation, and environmental uncertainty components remain unquantified.

## Data Availability

The raw data and processed data required to reproduce the findings of this study are not publicly available due to confidentiality restrictions of the cooperating institutions. Data are available from the corresponding author (Huiliang Cao) upon reasonable request, subject to the approval of Xi’an Microelectronics Technology Institute and Anhui XDLK Microsystem Corporation Limited.

## References

[1] Passaro V.M.N., Cuccovillo A., Vaiani L., De Carlo M., Campanella C.E. (2017). Gyroscope Technology and Applications: A Review in the Industrial Perspective. Sensors.

[2] Gill W.A., Howard I., Mazhar I., McKee K. (2022). A Review of MEMS Vibrating Gyroscopes and Their Reliability Issues in Harsh Environments. Sensors.

[3] Ru X., Gu N., Shang H., Zhang H. (2022). MEMS Inertial Sensor Calibration Technology: Current Status and Future Trends. Micromachines.

[4] Zhang H., Zhang C., Chen J., Li A. (2022). A Review of Symmetric Silicon MEMS Gyroscope Mode-Matching Technologies. Micromachines.

[5] Ren X., Zhou X., Yu S., Wu X., Xiao D. (2021). Frequency-Modulated MEMS Gyroscopes: A Review. IEEE Sens. J..

[6] Xu Y., Liu S., He C., Wu H., Cheng L., Yan G., Huang Q. (2024). Reliability of MEMS Inertial Devices in Mechanical and Thermal Environments: A Review. Heliyon.

[7] Peng T., You Z. (2021). Reliability of MEMS in Shock Environments: 2000–2020. Micromachines.

[8] Cao H., Cai Q., Zhang Y., Shen C., Shi Y., Liu J. (2021). Design, Fabrication, and Experiment of a Decoupled Multi-Frame Vibration MEMS Gyroscope. IEEE Sens. J..

[9] Sonmezoglu S., Alper S.E., Akin T. (2014). An Automatically Mode-Matched MEMS Gyroscope with Wide and Tunable Bandwidth. J. Microelectromech. Syst..

[10] Askari S., Asadian M.H., Shkel A.M. (2021). Performance of Quad Mass Gyroscope in the Angular Rate Mode. Micromachines.

[11] Chen C., Zhou J., Wang H., Fan Y., Song X., Xie J., Bäck T., Wang H. (2024). Machine Learning-Driven Discovery of High-Performance MEMS Disk Resonator Gyroscope Structural Topologies. Microsyst. Nanoeng..

[12] Cui M., Huang Y., Wang W., Cao H. (2019). MEMS Gyroscope Temperature Compensation Based on Drive Mode Vibration Characteristic Control. Micromachines.

[13] Zhang H., Chen W., Yin L., Fu Q. (2023). Design of MEMS Gyroscope Interface ASIC with On-Chip Temperature Compensation. Measurement.

[14] Li D., Zhou J., Liu Y. (2021). Recurrent-Neural-Network-Based Unscented Kalman Filter for Estimating and Compensating the Random Drift of MEMS Gyroscopes in Real Time. Mech. Syst. Signal Process..

[15] Li A., Cui K., An D., Wang X., Cao H. (2024). Multi-Frame Vibration MEMS Gyroscope Temperature Compensation Based on Combined GWO-VMD-TCN-LSTM Algorithm. Micromachines.

[16] Huang N.E., Shen Z., Long S.R., Wu M.C., Shih H.H., Zheng Q., Yen N.C., Tung C.C., Liu H.H. (1998). The empirical mode decomposition and the Hilbert spectrum for nonlinear and non-stationary time series analysis. Proc. R. Soc. Lond. A.

[17] Bie X., Xiong X., Wang Z., Yang W., Li Z., Zou X. (2023). Analysis of the Thermally Induced Packaging Effects on the Frequency Drift of Micro-Electromechanical System Resonant Accelerometer. Micromachines.

[18] Guo K., Wu Y., Zhang Y., Sun J., Xiao D., Wu X. (2020). Damping Asymmetry Trimming Based on the Resistance Heat Dissipation for Coriolis Vibratory Gyroscope in Whole-Angle Mode. Micromachines.

[19] Li J., Broas M., Makkonen J., Mattila T.T., Hokka J., Paulasto-Kröckel M. (2014). Shock Impact Reliability and Failure Analysis of a Three-Axis MEMS Gyroscope. J. Microelectromech. Syst..

[20] Xu Y., Lin J., He C., Wu H., Huang Q., Yan G. (2024). Design of a Shock-Protected Structure for MEMS Gyroscopes over a Full Temperature Range. Micromachines.

[21] Lian J., Li J., Xu L. (2019). The Effect of Displacement Constraints on the Failure of MEMS Tuning Fork Gyroscopes under Shock Impact. Micromachines.

